# The relevance of migraine in the clinical spectrum of mitochondrial disorders

**DOI:** 10.1038/s41598-022-08206-z

**Published:** 2022-03-10

**Authors:** Alberto Terrin, Luca Bello, Maria Lucia Valentino, Leonardo Caporali, Gianni Sorarù, Valerio Carelli, Ferdinando Maggioni, Massimo Zeviani, Elena Pegoraro

**Affiliations:** 1 Department of Neuroscience, ERN Neuromuscular Center, University of Padova, via Giustiniani, 5, 35128 Padua, Italy; 2 Department of Neurosciences, University of Padova, Padua, Italy; 3 Programma di Neurogenetica, IRCCS Istituto delle Scienze Neurologiche di Bologna, Bologna, Italy; 4 Department of Biomedical and Neuromotor Sciences (DIBINEM), University of Bologna, Bologna, Italy

**Keywords:** Migraine, Neuromuscular disease

## Abstract

Recent scientific evidence suggests a link between migraine and brain energy metabolism. In fact, migraine is frequently observed in mitochondrial disorders. We studied 46 patients affected by mitochondrial disorders, through a headache-focused semi-structured interview, to evaluate the prevalence of migraine among patients affected by mitochondrial disorders, the possible correlations between migraine and neuromuscular genotype or phenotype, comorbidities, lactate acid levels and brain magnetic resonance spectroscopy. We explored migraine-related disability, analgesic and prophylactic treatments. Diagnoses were achieved according to International Classification of Headache Disorders, 3rd edition. Lifetime prevalence of migraine was 61% (28/46), with high values in both sexes (68% in females, 52% in males) and higher than the values found in both the general population and previous literature. A maternal inheritance pattern was reported in 57% of cases. MIDAS and HIT6 scores revealed a mild migraine-related disability. The high prevalence of migraine across different neuromuscular phenotypes and genotypes suggests that migraine itself may be a common clinical manifestation of brain energy dysfunction. Our results provide new relevant indications in favour of migraine as the result of brain energy unbalance.

## Introduction

Migraine is the second leading cause of years lived with disability in the world (Global Burden of Disease Study)^1^. Its one-year prevalence in women and men is 18% and 6%, respectively, with prevalence peaks between the ages of 25 and 55^2–4^. Migraine is associated with a considerable financial burden^5,6^ and its impact on functioning can be evaluated using validated scales as Headache Impact Test (HIT6)^7^, Migraine Disability Assessment (MIDAS)^8^ or the recent Migraine Physical Function Impact Diary^9^. The last decades witnessed a solid progress in understanding the mechanisms underpinning migraine, but much still remains to be explained^10^. A pioneering view of primary headaches indicates them as “adaptive behavioural responses”^11^. Brain ^31^P-magnetic resonance spectroscopy (MRS) findings and mitochondrial energy metabolism markers in blood and muscle supported this theory^12,13^. In confirmation of this idea, Lisicki and collaborators suggested an activity-induced rupture of cerebral metabolic homeostasis in migraineurs, using ^18^FDG-PET and visual evoked potentials^14^. A recent review pointed to a role of energy metabolism in the pathogenesis of migraine, with mitochondria carrying out a central role^15^. Some authors speculate that migraine is a “conserved and adaptive response that occurs in genetically predisposed individuals with a mismatch between the brain energy reserve and workload”^15^. Under this assumption, we evaluated the prevalence of primary headaches and, in particular, of migraine (migraine without aura (MO), migraine with aura (MA), hemiplegic migraine (HM), chronic migraine (CM), probable migraine without aura (pMO) or probable migraine with aura (pMA)) among patients suffering from mitochondrial disorders, looking for clinical hints supporting the relationship between migraine and energy metabolism.

## Results

### Prevalence of primary headaches among patients with mitochondrial disorders

Forty-six individuals affected by mitochondrial disorders were included with a mean age of 55 ± 14.6 years (range 17–83 years), 25 females (54.3%) and 21 males (45.7%). Body Mass Index (BMI) ranged from 15.2 to 30.8 kg/cm^2^ in females (mean value 22.1 ± 4.2 kg/cm^2^) and from 16.7 to 34.0 kg/cm^2^ in males (mean value 24.1 ± 4.3 kg/cm^2^). Other demographic features and cardiovascular risk factors are reported in Table 1. In this context, “Stroke/TIA” refers to an actual cerebrovascular events, i.e.: stroke in case of clear ischemic bran lesions, and TIA in case of transient acute neurological deficit in patients with multiple cerebrovascular risk factors.

**Table 1:** Demographic features and cardio-cerebrovascular risk factors distribution in 46 patients, distinguished by sex.

	Females	Males
Population	25 (54.3%)	21 (45.7%)
Age	53.9 ± 15.8	56.4 ± 13.4
Weight (kg)	57.1 ± 11.7	73.9 ± 15.3
Height (cm)	160.7 ± 5.6	174.6 ± 7.0
BMI (Kg/cm^2^)	22.1 ± 4.2	24.1 ± 4.3
Married	17/25 (68.0%)	13/21 (61.9%)
Primary school	1/25 (4.0%)	3/21 (14.3%)
Middle school	9/25 (36.0%)	6/21 (28.6%)
High school	8/25 (32.0%)	7/21 (33.3%)
Graduated	5/25 (20.0%)	4/21 (19.0%)
Smokers	8/25 (32.0%)	9/21 (42.9%)
Hypertension	7/25 (28.0%)	10/21 (47.6%)
Dyslipidaemia	7/25 (28.0%)	6/21 (28.6%)
Diabetes mellitus	3/25 (12.0%)	4/21 (19.0%)
CHD	2/25 (8.0%)	2/21 (9.5%)
Stroke/TIA	3/25 (12.0%)	2/21 (9.5%)
Alcohol	14/25 (56.0%)	13/21 (61.9%)
Hormonal contraceptives	13/25 (52.0%)	/

*CHD* coronary heart disease.

The interview was conducted face-to-face in 57% of cases (26/46), and by telephone in 43% (20/46). A total of 21/46 patients (45.7%) reported at least one first-grade or second-grade relative who had suffered from recurrent headaches. In this group, a maternal inheritance pattern was reported in 12/21 cases (57%); paternal inheritance in two cases, while siblings but not parents were affected in the remaining seven cases (33%).

**Figure 1:**
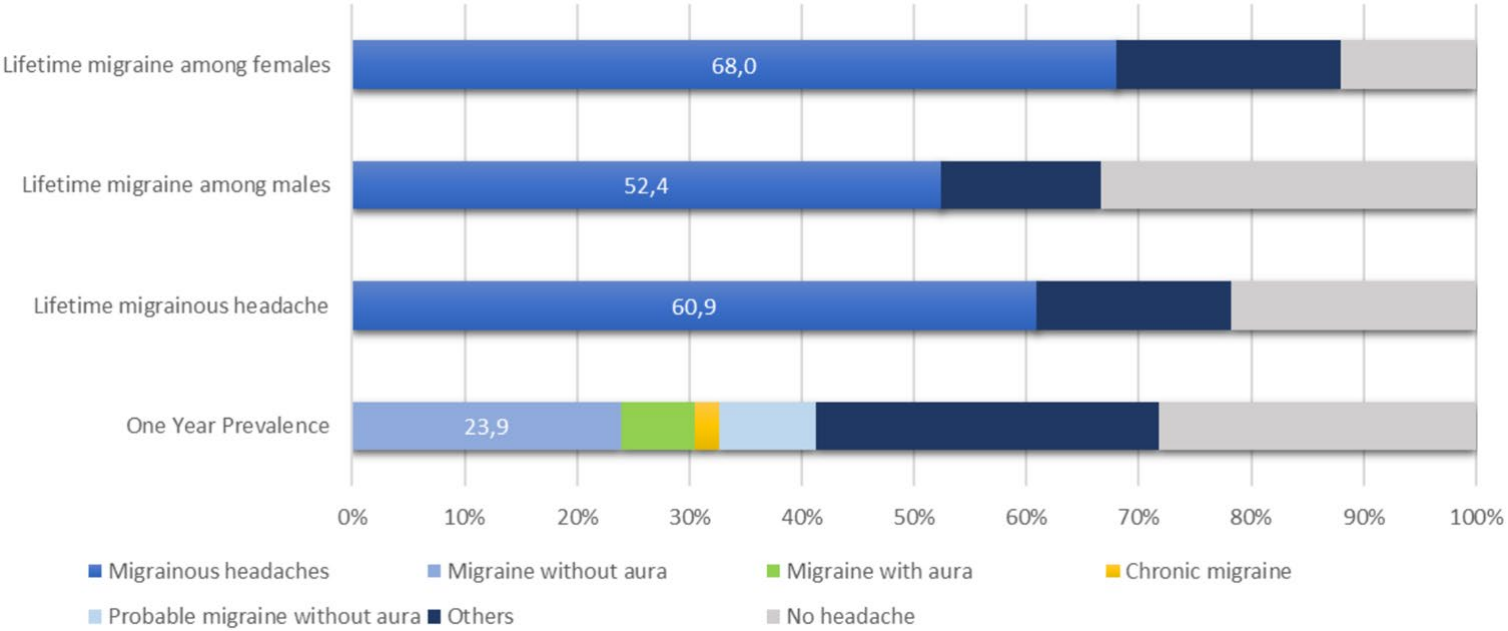
Prevalence of primary headaches among patients with mitochondrial disorders, according to ICHD3 criteria. One-year prevalence (lower bar); lifetime prevalence of migrainous headache (including migraine without aura, migraine with aura, chronic migraine and probable migraine without aura) by sex (first and second bars) and in the whole study population (third bar).

According to ICHD3 criteria, we considered first the one-year prevalence: 28.3% of our patients (13/46) reported no headache in the last year, 11 patients (23.9%) suffered from MO, one had CM and 4/46 (8.7%) had pMO. MA was reported in 2/46 (4.3%) and one had sporadic HM. Aggregate data show 41.3% of patients (19/46) suffering of headache with migrainous features in the last year. A group of 7/46 patients (15.2%) reported infrequent episodic tension-type headache (ETTH), 5/46 (10.9%) had frequent ETTH, and one had post-ictal headache and one unspecified headache (Fig. 1).

Considering the wide age range (17–83 years) and the high mean age of our patients cohort, data about the lifetime prevalence of migraine are more relevant: 18/46 (39.1%) of patients achieved the diagnosis of MO (in one case together with MA diagnosis), one reported CM, 2/46 (4.3%) had MA, in one case with hemiplegic features. A group of 7/46 patients (15.2%) reported pMO. Among the pMO patients, diagnostic criterion D (“during headache at least one of the following: 1. Nausea and/or vomiting; 2. Photophobia and phonophobia”^16^) was lacking in four cases. Criteria B (“headache attacks lasting 4–72 h, when untreated or unsuccessfully treated”^16^) and C (“headache has al least two of the following four characteristics: 1. Unilateral location, 2. Pulsating quality, 3. Moderate or severe pain intensity, 4. Aggravation by or causing avoidance of routine physical activity”^16^) were not satisfied in two and one cases, respectively. Overall, 60.9% (28/46) of patients with a mitochondrial disorder suffered from a migrainous headache in their lifetime. Migraine prevalence was 68.0% in females (17/25), and 52.4% (11/21) in male patients (Fig. 1). Only 10/46 patients (21.7% of cases) did not report any sort of recurrent headache in their life, despite a higher mean age (66.0 ± 13.9 years; range 42–83 years).

### Clinical features of headache in mitochondrial patients

**Figure 2:**
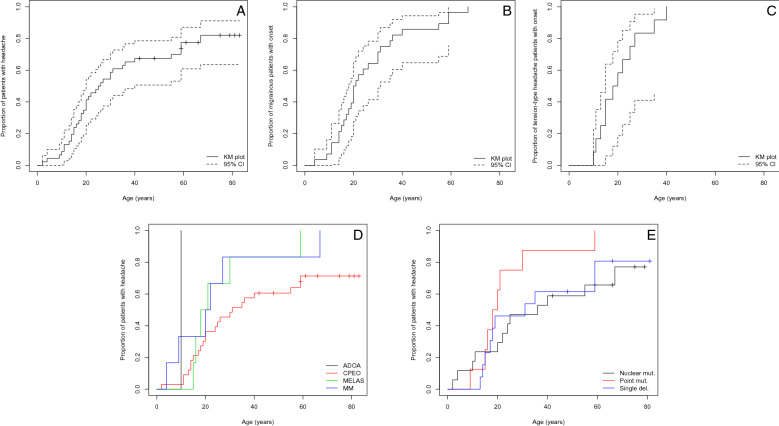
(**A**): Cumulative incidence plot of headache with increasing age. Crosses indicate censored patients. (**B**): Cumulative incidence plot of migraine, with increasing age. (**C**): Cumulative incidence plot of tension-type headache, with increasing age. (**D**): Cumulative incidence plot of headache with increasing age, according to different mitochondrial genotypes. (**E**): Cumulative incidence plot of headache with increasing age, according to different neuromuscular phenotypes. Dashed lines indicate the 95% confidence interval. Each cross indicates censored patients. Abbreviations: ADOA = autosomal dominant optic atrophy; CPEO = chronic progressive external ophthalmoplegia; MELAS = mitochondrial encephalopathy, lactic acidosis and stroke-like episodes; MM = other mitochondrial myopathy.

The median age at onset of headaches was 25.5 years, with 95% confidence interval from 20 to 40 years (Fig. 2A); this model includes “censored” patients who never developed headache. Migraine showed a median clinical onset at about 20 years, where TTH patients had an earlier median onset, at 19 years (Fig. 2B,C); these headache-type-specific models excluded “censored” patients, hence the earlier estimated median values. The difference between the median age at onset among the different headache subtypes was not statistically significant. Nine patients reported the past resolution of a highly-disabling migrainous headache towards a less impacting ETTH (5 cases) at the mean age of 44 years or a complete resolution of their MO (2 cases) or pMO (1 case), at a mean age of 51 years. One patient reverted from CM to MO, while another one stopped suffering from MO, while continuing with his MA attacks.

Aura prevalence reported in only 3 patients (10.7% of the migraineurs): 2 were typical auras, while 1 patient carrying *MICU1* mutations experienced hemiplegic migraine.

Our 28 migrainous patients had experienced, at least one, 64% (42/66) of the triggers listed in our semi-structured 66-item-questionnaire, including the most frequent triggers of migrainous headache, according to specific scientific literature^17–19^. Each patient reported up to 15 different triggers of migraine. As expected, among the most reported factors there were stress (39%), work-related stress (36%) or family-related stress (29%). More frequently, 54% of migrainous patients could had attacks triggered by sleeping deprivation, 11% with sleeping more than usual or 18% in case of sleeping in the afternoon. 53% of female migraineurs pointed to female hormonal fluctuations (including premenstrual period, menstrual phase, ovulatory period or oral contraceptives) (see Table 2 for the complete list).

**Table 2:** Trigger factors for migraine attacks.

Trigger	Prevalence (%)	Prevalence (n)	N ref
Lack of sleep	53.6	15	28
Anxiety	42.9	12	28
Premenstrual period	41.1	7	17
Unspecified stress	39.3	11	28
Anger	35.7	10	28
Work-related stress	35.7	10	28
Warm days	28.6	8	28
Family-related stress	28.6	8	28
Menstrual period	23.5	4	17
Sun exposure	21.4	6	28
Strenuous physical exercise	21.4	6	28
Intense lights	21.4	6	28
Physical fatigue	17.9	5	28
Daytime sleep	17.9	5	28
Windy days	17.9	5	28
Wet days	17.9	5	28
Fasting/skipping meals	14.3	4	28
Cold days	14.3	4	28
Other	14.3	4	28
Ovulatory period	11.7	2	17
Sleeping more than usual	10.7	3	28
Loud noises	10.7	3	28
Fever	10.7	3	28
Cigarette smoke	10.7	3	28
Relaxing after stressful period	10.7	3	28
Binge eating	10.7	3	28
Other climate changes	10.7	3	28
Scents	10.7	3	28
Other emotional reaction	7.1	2	28
Mood changes	7.1	2	28
Aeroplane travels	7.1	2	28
Other food	7.1	2	28
Oral contraceptives	5.9	1	17
Red wine	3.6	1	28
Dehydration	3.6	1	28
Hypoglycaemia	3.6	1	28
High-altitude	3.6	1	28
Long travels	3.6	1	28
White wine	3.6	1	28
Other alcoholics	3.6	1	28
Fruits	3.6	1	28
Vegetables	3.6	1	28

Complete list of the triggers reported by patients suffering from a migrainous headache; triggers have been selected among the items listed in our semi-structured questionnaire. N. ref. = number of patients representing the total for each item.

The quality of pain among the 28 migraineurs was throbbing in 64% (18/28) of cases, with a mean headache intensity of 8.25/10 (Number Rating Scale). It was reported as unilateral in 46% (13/28) of cases, bilateral in 54% (15/28), with predominant origin in the frontal area (36%–10/28) and irradiation to the homolateral half of the head (21%–6/28). Moderate physical activity exacerbated headache in 79% (22/28) of cases. Prodromal symptoms were reported by only 3 MO patients (11% of migraineurs), and included tiredness, neck stiffness, fatigue, confusion and blurred vision. Accompanying symptoms consisted of nausea (79%–22/28), vomiting (50%–14/28), photophobia (79%–22/28), phonophobia (54%–15/28) and osmophobia (18%–5/28). Allodynia was reported by 18% (5/28) of migrainous cohort too, with a tendency towards a correlation with a higher number of migraine attacks per month: a mean of 9.2 ± 13.1 attacks per month among allodynic patients, versus a mean of 3.2 ± 3.8 attacks per month among non-allodynic migraineurs (*p* = 0.093) (Supplementary Information 1).

Migrainous attacks had a mean duration of 24.1 ± 25.1 h. At the time of the interview, patients reported a mean frequency of 4.1 ± 6.1 migraine attacks per month (range 0.3–30), with a median value of 2.5 attacks per month. 12 patients (43%) reported more than three migraine attacks per month that were often moderately or severely disabling (75% of these sub-sample). Two other patients reported a mean of three highly disabling migraine attacks per month, whose duration may reach the 48 h. 50% of our active migraineurs did benefit from a prophylactic therapy, but none was regularly assuming it. Considering the whole sample, only 18% (5/28) of our migrainous patients had tried a preventive therapy at least once in their life, including flunarizine (3 cases), followed by topiramate, pizotifen, propranolol, acupuncture and nutraceutical (1 case each).

### Mitochondrial phenotype and genotype and migraine

**Figure 3:**
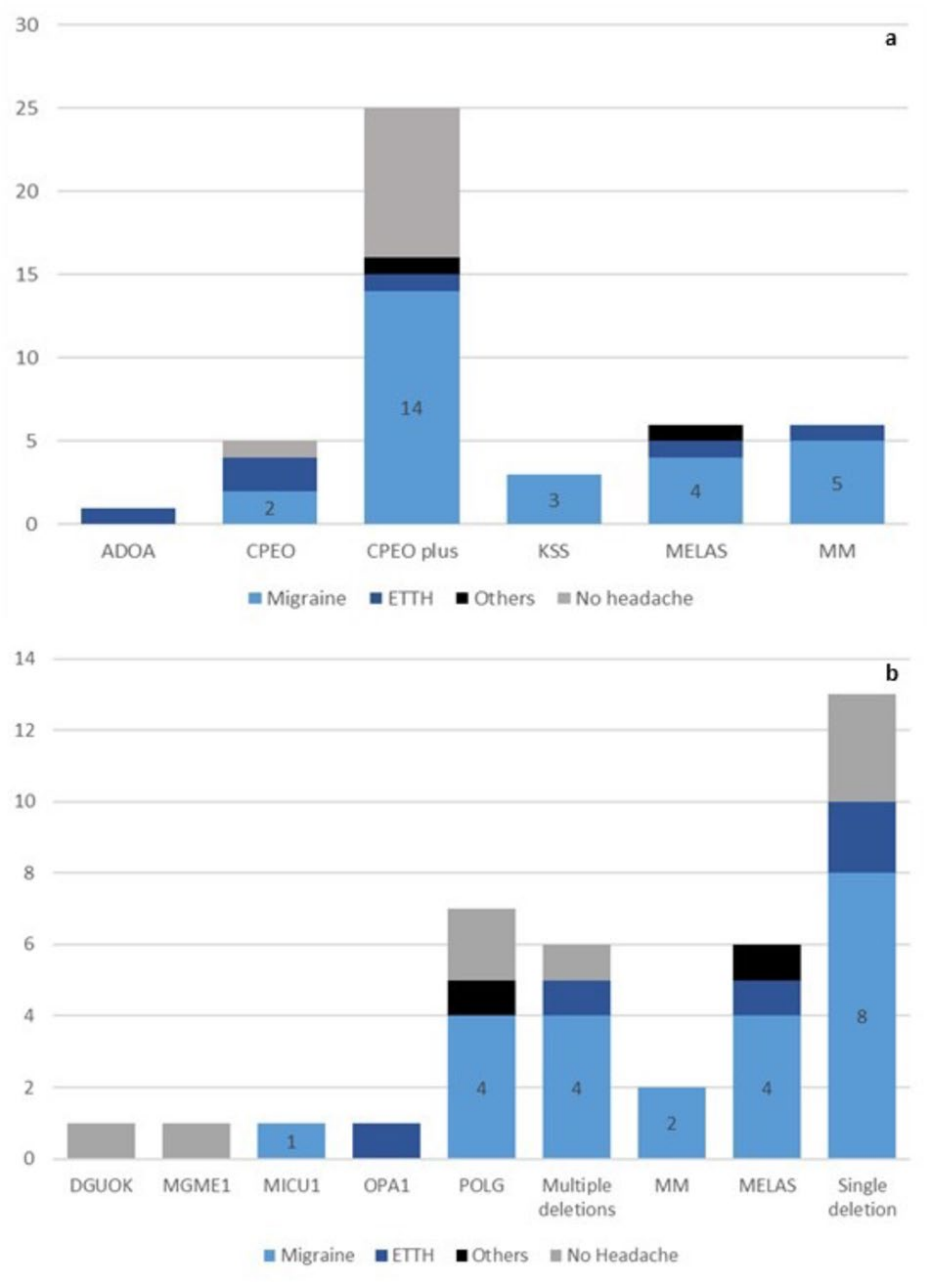
Histograms showing the distribution of migraine, ETTH, other types of headache or absence of headache in all phenotypic (**a**) and genotypic (**b**) groups of mitochondrial disorders. Legend: light blue = includes migraine without aura, migraine with aura, hemiplegic migraine, chronic migraine, probable migraine without or with aura; dark blue = episodic tension-type headache, black = other types of headache; grey = no headache sufferers. Abbreviations: ADOA = autosomal dominant optic atrophy; CPEO = chronic progressive external ophthalmoplegia; KSS = Kearns-Sayre syndrome; MELAS = mitochondrial encephalopathy, lactic acidosis and stroke-like episodes; MICU1 = mitochondrial calcium uptake 1; MM = other mitochondrial myopathy. DGUOK = deoxyguanosine kinase; MELAS = mitochondrial encephalopathy, lactic acidosis and stroke-like episodes; MGME1 = mitochondrial genome maintenance exonuclease 1; MICU1 = mitochondrial calcium uptake 1; MM = other point mtDNA mutations; multiple deletions: multiple mtDNA deletions; OPA1 = OPA1 mitochondrial dynamin like GTPase; POLG = DNA polymerase gamma; single deletion = single mtDNA deletion.

Clinical phenotype (Table 3) was consistent with CPEO in 33 patients (5 CPEO, 25 CPEO plus and 3 KSS), MELAS in 6, ADOA in 1, other MM in 6 patients (a young patient with *MICU1* gene mutation and other unspecified MM in 5 cases) (Table 4). 19/33 CPEO patients (58%) suffered from a migrainous headache: 12 MO (36%), 1 CM and 6 pMO. All 10 patients who denied the experience of recurrent headaches in their medical history belonged to CPEO subgroup. 67% of MELAS patients were migraineurs (2/6 MO, 1/6 MA, 1/6 MO plus MA), while 83% of patients with other MM reported a migrainous headache (3/6 MO, 1 pMO, 1 HM). The only ADOA patient had a ETTH (Table 5, Fig. 3a). The absence of any primary headaches results associated with CPEO phenotype with statistical significance (*p* = 0.036). The age of onset of headache was not significantly different across various phenotypic groups (Fig. 2d).

**Table 3:** Summary of main clinical phenotypes of mitochondrial disorders, with relative clinical clues and genotypes.

TAG	Phenotype	Clinical features	Genotype
CPEO	Chronic Progressive External Ophthalmoplegia	Progressive bilateral ptosis and diffuse reduction in ocular motility	Large-scale mtDNA deletions OR multiple mtDNA deletions (due to mutations in *TWNK*, *POLG*, *TK2*, *RRM2b*, *DGUOK* genes or others) OR point mtDNA mutations
CPEO plus	Chronic Progressive External Ophthalmoplegia Plus	Progressive bilateral ptosis and ophthalmoparesis associated to multiple features of neuromuscular and multisystem involvement	See “CPEO” Genotype
KSS	Kearns-Sayre syndrome	Early onset CPEO with cardiac conduction block and pigmentary retinopathy, w/o multisystem involvement; Ataxia, Psychomotor regression	Large-scale mtDNA deletions
ADOA	Autosomal Dominant Optic Atrophy	Visual loss starting during 1st decade of life, color vision defects	nDNA mutations (*OPA1*–*OPA3*–*OPA 4–5-8* gene)
MELAS	Mitochondrial Encephalomyopathy Lactic Acidosis and Stroke-like episodes	Seizures, Ataxia, Myoclonus, Psychomotor regression, Cortical blindness, Dystonia, Weakness, Sensorineural hearing loss, Short stature, Lactic acidosis, hemiparesis/hemianopia	tRNA point mutation (m.3243A > G)
MM	Other Mitochondrial Myopathy	All other combinations of muscle weakness w/o multisystem involvement	nDNA mutations OR mtDNA depletions OR mtDNA deletions

**Table 4:** Study population divided by neuromuscular phenotype (rows) and genotype (columns).

Phenotype	Genotype										
	nDNA					Likely nDNA	mtDNA point		mtDNA deletion	N/A	TOT
	*DGUOK*	*MGME1*	*MICU1*	*OPA1*	*POLG*	Multiple deletions	mtM	MELAS	Single deletion		
ADOA				1							1
**CPEO**											
CPEO						2			3		5
CPEO Plus	1	1			7	2			7	7	25
KSS									3		3
MELAS								6			6
MM			1			2	2			1	6
TOT	1	1	1	1	7	6	2	6	13	8	46

*ADOA* autosomal dominant optic atrophy, *CPEO* chronic progressive external ophthalmoplegia, *KSS*Kearns-Sayre syndrome, *MELAS*mitochondrial encephalopathy, lactic acidosis and stroke-like episodes, *MICU1* mitochondrial calcium uptake 1, *MM*other mitochondrial myopathy, *DGUOK*deoxyguanosine kinase, *MGME1* mitochondrial genome maintenance exonuclease 1, *MICU1*mitochondrial calcium uptake 1, *OPA1*OPA1 mitochondrial dynamin like GTPase, *POLG*DNA polymerase gamma, *mtM* other point mtDNA mutations, *MELAS* mitochondrial encephalopathy, lactic acidosis and stroke-like episodes.

**Table 5:** Distribution of ICHD3 diagnoses with different mitochondrial phenotypes.

Phenotype	No	MO	MA/HM	CM	pMO	iETTH	fETTH	Others	Tot
ADOA	0	0	0	0	0	0	1	0	1
CPEO	1	0	0	0	2	2	0	0	5
CPEO plus	9	9	0	1	4	1	0	1	25
KSS	0	3	0	0	0	0	0	0	3
MELAS	0	3	1	0	0	0	1	1	6
MICU1	0	0	1	0	0	0	0	0	1
MM	0	3	0	0	1	1	0	0	5
Tot	10	18	2	1	7	4	2	2	46

*No* no headache, *MO* migraine without aura, *MA* migraine with aura, *HM* hemiplegic migraine, *CM* chronic migraine, *pMO* probable migraine without aura, *iETTH* infrequent tension-type headache, *fETTH* frequent tension-type headache. *ADOA* autosomal dominant optic atrophy, *CPEO* chronic progressive external ophthalmoplegia, *KSS* Kearns-Sayre syndrome, *MELAS* mitochondrial encephalopathy, lactic acidosis and stroke-like episodes, *MICU1* mitochondrial calcium uptake 1, *MM* other mitochondrial myopathy.

A total of 32/46 patients (70%) had a defined genotype. 11 patients harboured nDNA mutations and represented the largest group: *POLG* mutations were identified in 7, and *OPA1*, *DGUOK*, *MGME1* and *MICU1* gene mutation in one patient each. Six patients had multiple mtDNA deletions but no nuclear gene mutation identified yet. Thirteen patients showed a single mtDNA deletion, while eight had a mtDNA point mutation: A3243G in 6, a tRNA_Leu_ (mt.C3250T) and tRNA_Met_ (mt.A4415G) in in one patient each. 60.5% (23/38) of patients with a defined disease-causing mutation or with multiple mtDNA deletions suffered from a migrainous headache. Among patients with nDNA mutations, migrainous headache (4 MO, 4 pMO and one HM) was present in 52.9% of patients (9/11). Sixty-two percent (8/13) of patients with single mtDNA deletion were migraineurs (6 MO and 2 pMO). The mtDNA point mutations were associated with migraine in 75% of cases (6/8): 5 MO and one MA (Table 6, Fig. 3b). The distribution of primary headaches with the different genotypes did not differ significantly among subgroups. Age at headache onset and lack of primary headaches was not statistically different according to genotypes (Fig. 2e).

**Table 6:** Distribution of ICHD3 diagnoses with different mitochondrial genotypes.

	Genotype	No	MO	MA/HM	CM	pMO	pMA	iETTH	fETTH	Others	Tot
nDNA	DGUOK	1	0	0	0	0	0	0	0	0	1
	MGME1	1	0	0	0	0	0	0	0	0	1
	MICU1	0	0	1	0	0	0	0	0	0	1
	OPA1	0	0	0	0	0	0	0	1	0	1
	POLG	2	2	0	0	2	0	0	0	1	7
Likely nDNA	Multiple deletions	1	2	0	0	2	0	1	0	0	6
mtDNA point	mtM	0	2	0	0	0	0	0	0	0	2
	MELAS	0	3	1	0	0	0	0	1	1	6
mtDNA deletion	Single deletion	3	6	0	0	2	0	2	0	0	13
Total	All	8	15	2	0	6	0	3	2	2	38

*No* no headache, *MO* migraine without aura, *MA* migraine with aura, *HM* hemiplegic migraine, CM chronic migraine, *pMO* probable migraine without aura, *pMA* probable migraine with aura, *iETTH* infrequent tension-type headache, *fETTH* frequent tension-type headache, *DGUOK* deoxyguanosine kinase, *MGME1* mitochondrial genome maintenance exonuclease 1, *MICU1* mitochondrial calcium uptake 1, *OPA1* OPA1 mitochondrial dynamin like GTPase, POLG DNA polymerase gamma, *mtM* other point mtDNA mutations, *MELAS* mitochondrial encephalopathy, lactic acidosis and stroke-like episodes.

Among 19 patients with active migraine, mean HIT6 score was 55.1 ± 6.8 pts (range 46–65). Eleven patients reported mild (HIT6 score between 50 and 55 points) or no disability (HIT6 score < 50 points) related to their migraine; eight (42%) experienced a moderate (HIT6 score from 56 to 59 points) or severe (HIT6 score > 59 points) migraine-related disability. CPEO plus patients showed a tendency towards a higher degree of disability and KSS patients reported a mild degree of disability, but no statistically significant differences in HIT6 scores could be found across different phenotypes or genotypes. *POLG* patients showed a tendency towards a higher migraine-related disability (Supplementary Information 2).

The 19 patients with active migraine reported a mean MIDAS score of 11.2 ± 19.6 points (range 0–80 points) with a median of 1 point. A mild (MIDAS score from 6 to 10 points) or neglectable migraine-related disability (MIDAS score < 6 points) has been reported by 74% (14/19) of the patients, with only two cases of moderate disability (MIDAS score from 11 to 20 points) and three patients with severe disability related to migraine (MIDAS score > 20 points). The last five patients had a moderate-severe disability score at HIT6, as well.

#### Treatment strategies

the great majority of patients (79%–22/28) took non-steroidal anti-inflammatory drugs (NSAIDs), with paracetamol and ibuprofen as the leading drugs (29% of cases, each), combination-analgesic or metamizole in three cases, while three patients (11%) did not assume any acute treatment for their migraine attacks. None of our 28 mitochondrial patients treated their migraine with triptans or ergot alkaloids, even if two patients (7% of migraineurs) reported previous Medication Overuse Headache (one paracetamol-overuse headache and a combination-analgesic overuse headache).

**Figure 4:**
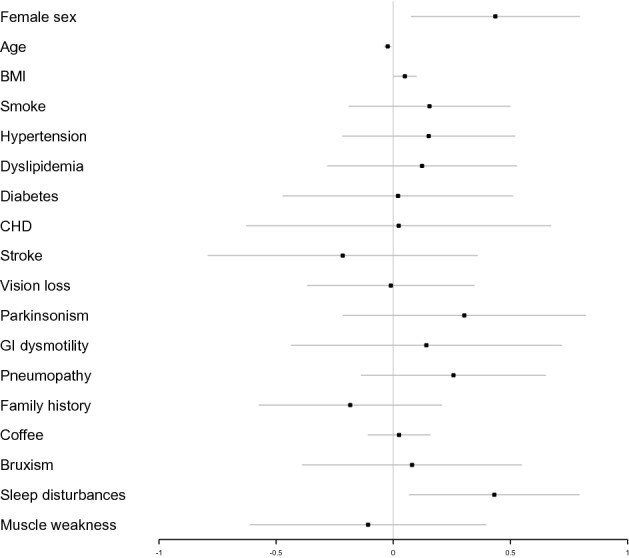
Forest plot showing the odds ratios, confidence intervals 95% and direction of association between migraine and demographic features, cardiovascular risk factors and other clinical conditions. Multivariate analysis showed that female sex, age, and sleep disturbances were significantly associated with the diagnosis of migraine.

A logistic regression analysis of risk of the onset of migraine was performed including variables such as sex, age, BMI, cardiovascular risk factors, vision loss, parkinsonism (defined as the presence of clinical signs suggesting a dysfunction of the extrapyramidal system), gastrointestinal dysmotility, respiratory involvement, family history, coffee use, bruxism, sleep disturbances (including snoring, obstructive sleep apnoea or insomnia) and muscular weakness. This multivariate analysis showed that female sex (logistic regression coefficient 0.44, 95% CI 0.076–0.80, *p* = 0.025), increasing age (coefficient − 0.024, 95% CI − 0.039 ~ − 0.009, *p* = 0.005) and sleep disturbances (coefficient 0.431032, 95% CI 0.068–0.80, *p* = 0.029) were significantly associated with a lifetime diagnosis of migraine (Fig. 4). BMI showed a trend to association with migraine onset (coefficient 0.050, 95% CI 0.0007–0.099, *p* = 0.058).

Among possible biochemical markers, blood lactate was available only in 67% (31/46) of patients. The mean value was 2.5 ± 3.9 mmol/L (range 0.5–20.0 mmol/L; normal level < 2.2 mmol/L), with higher levels among the 17/31 migrainous patients (mean 3.5 ± 5.1 mmol/L; range 0.8–20.0 mmol/L) compared to the 14/31 non-migraineurs (mean 1.2 ± 0.7 mmol/L; range 0.5–2.5 mmol/L). Differences in blood lactate levels were statistically significant (*p* = 0.012 Supplementary Information 3). Seventeen patients (37%) had a brain ^1^H-MRS (as part of a different previous research protocol about mitochondrial disorders, independently from the presence/absence of headaches), in 3 cases (3/17–18%) revealing an increase in lactate levels and a migrainous headache (100%–3/3). A total of 43% of patients with a normal MRS study received a diagnosis of migraine (6/14); Fisher’s test was not significant.

## Discussion

Our 46 patients cross-sectional study represents the fourth largest study in scientific literature, regarding primary headaches in adult patients with mitochondrial disorders. We found a one-year prevalence of migrainous headache of 41%, significantly greater than the prevalence reported in general population in the European countries^20,21^, and higher than the 25.9% prevalence reported in a recent large Italian population study^22^. A total of 61% of mitochondrial disease patients suffered or had suffered of a migrainous headache. Since our sample has a high mean age and migraine prevalence is known to decrease with increasing age^20^, the prevalence in our patients’ cohort is more than threefold higher than expected in the general population^20^ in both sexes, reaching 68% in female. Our estimates are higher than those reported in a recent large Dutch study (55%, mainly focused on mt.3243A > G patients^23^) and in a previous study with more heterogenous subtypes of mitochondrial patients (35.5%^24^). Given the well-known high prevalence of migraine among mt.3243A > G patients^25,26^, the limited number of MELAS patients in our cohort minimizes selection bias. CPEO and CPEO plus are the main phenotypes in our study population, with a subgroup prevalence of migraine of 53%, which is notably higher than the 21–40% prevalence previously reported among CPEO patients^27–29^. We found higher migraine prevalence also in *POLG* patients (57 vs. 23%)^30^, whereas the prevalence was similar to that of literature in MELAS patients (67%)^26^. Age at onset, evolution over time, prodromal symptoms, trigger, features and duration of headache are in line with those of the general population with migraine, confirming that the clinical entity is likely the same. However, a few differences from the general population emerged. First, phonophobia shows 54% prevalence and osmophobia involves only 18% of migraineurs, which are frequencies lower than those literature ^17,31^. This could be partially due to the frequent hearing loss (reported up to 43% of our cohort) or to the decrease in olfactory perception induced by mitochondrial pathology (nearly 10% of our sample). In fact, in our 7 pMO patients the diagnostic criterion D (“During headache at least one of the following: (1) nausea and/or vomiting; (2) photophobia and phonophobia”^16^) was absent more often than other definitory features. Furthermore, the 11% prevalence of prodromal symptoms is significantly lower than the 72% reported in literature^10^, with a probable bias due to memory recall. These data suggest that we did not document a mere migraine-like headache: migraine, as coded in ICHD3, appears indeed a core clinical manifestation of the clinical spectrum of mitochondrial disorders.

Considering the familiar occurrence of migraine in general population, a maternal inheritance pattern is by far the most frequent^32,33^, providing another hint in favour of a possible role of mitochondria in migraine pathogenesis.

Interestingly, isolated stress (50%), lack of sleep (54%), oestrogen fluctuations (53%), fatigue (24%), vigorous physical exercise (18%), fasting or skipping meals (15%) are some of the consistently reported migraine triggers which can be connected to failure of energy metabolism, mitochondrial dysfunction and oxidative stress. Accordingly, and in contrast with previous literature data^24^, we observed a statistically significant association between migraine and increased blood lactate levels, likewise our 3 MO patients all had increased lactate levels detected by ^1^H-MRS.

We failed to find any significant difference in age at onset and migraine prevalence, nor was migraine prevalence significantly different across various mitochondrial genotypes, with a prevalence ranging from 53% (nDNA mutation) to 75% (mtDNA point mutation). This lack of correlation between migraine and neuromuscular phenotype or genotype is in line with previous literature^24^ and suggests that migraine is likely associated with dysfunction of brain energy metabolism, rather than with a specific mutation or clinical pattern.

Migraine showed a statistically significant association with female sex and sleep disturbances (and a tendency towards a correlation with increasing BMI), in accordance with previous studies on migraine^34–37^, strengthening the theorized central role of energy unbalance as the fulcrum of migraine pathogenesis.

Concerning the the results of HIT6 and MIDAS scales, the mean values reported by the only previous work on migraine in mitochondrial disorders^23^ point to a mild level of disability. In our study, 42% of our active-migraine individuals reported an at least moderate migraine-related impairment on HIT6 scale, compared to the 62% prevalence found by Tiehuis and collaborators in a younger Dutch population^23^. 26% of our patients had a disabling migraine according to MIDAS, versus 34% in the scientific literature^23^. No association between HIT6/MIDAS scores and mitochondrial phenotypes or genotypes has been found. However, “CPEO plus” and *POLG* patients showed a tendency towards a higher degree of disability.

Data on migraine treatment were surprising. None of our patients was being administered a preventive therapy and only 18% of patients with a lifetime diagnosis of migrainous headache had tried prophylaxis before our examination, nor acute treatment was previously considered: no one had tried triptans in their life, whereas common analgesics were commonly used in a few cases. Treatment of migraine was far less than optimal in patients with mitochondrial disorders, often without exploiting the most appropriated available pharmaceutical tools. This under-treatment of migraine is in line with the few available data in scientific literature^23^: it could be partially due to the (wrong) underevaluation, both by patients and physicians, of the role played by headache in the syndromic output of the patients, when compared to the systemic and sometimes life-threatening dysfunctions associated with myopathy or mitochondrial pathology.

Our study sheds light on the role of migraine in mitochondrial diseases, with some limitations. First, the cross-sectional design of our study may introduce a recall bias. Second, the still limited size of our cohort of patients makes it difficult to reach statistically sound associations between migraine and neuromuscular phenotypes or genotypes. Third, we lack a matched control group for a direct comparison. Fourth, we did not perform any measure of cognitive and/or memory performance in order to quantify the consistency and completeness of reported data, and finally, psychiatric comorbidities have not been considered in our study.

In this observational study, our preliminary clinical data converge to define migraine and its subtypes as a typical clinical manifestation of mitochondrial disorders. The lifetime prevalence of migraine was 61%, which is markedly higher than the values found in both the general population and the previous literature concerning mitochondrial disorders. In agreement with a previous article ^24^, the prevalence of migraine was high across different neuromuscular phenotypes and genotypes suggesting that migraine itself may be a common clinical manifestation of brain energy dysfunction. Our results provide new relevant indications in favour of migraine as the result of brain energy unbalance.

## Materials and methods

### Ethical approval

This study was approved by the “Ethical Committee for Clinical Studies of the Padova district “ and have therefore been performed in accordance with the ethical standards laid down in the 1964 Declaration of Helsinki and its later amendments.

### Patients

We performed an observational study on a series of patients affected by mitochondrial disorders followed at the Neuromuscular Center of the Neurological Clinic of the University of Padova. We included patients with a genetically confirmed diagnosis of mitochondrial disorder and patients in whom the combination of clinical (muscle w/o multisystem involvement), biochemical (OXPHOS dysfunction), neuroimaging (MR abnormalities) and histopathological (ragged red fibers, COX negative/SDH positive fibers, subsarcolemmal rims at SDH) data could strongly suggest the diagnosis of mitochondrial encephalomyopathy, even in absence of a known pathogenic mutation. Patients were clinically classified in “CPEO” (progressive bilateral ptosis and ophthalmoparesis), “KSS” (Kearns-Sayre syndrome, early onset CPEO plus cardiac conduction block and pigmentary retinopathy w/o multisystem involvement), “CPEO plus” (CPEO associated to multiple features of neuromuscular and multisystem involvement), autosomal dominant optic atrophy (“ADOA”), mitochondrial encephalomyopathy lactic acidosis and stroke-like episodes (“MELAS”) and other mitochondrial myopathy (“MM”). Patients were genotypically classified in individuals carrying nuclear DNA (nDNA) mutations, mitochondrial DNA (mtDNA) mutations and accumulation of multiple mtDNA deletions. All patients underwent neurological examination. Brain MRS and blood lactate levels were collected in a subset of patients.

### Headache evaluation

A semi-structured headache-focused interview was performed by a neurologist with specific training in the headache field; whenever possible it was carried out face-to-face, otherwise a telephone interview was performed. We asked for relatives suffering from headache, the time of onset of headache in the personal medical history and its evolution over time, the prodromal and associated symptoms, possible triggers (through a detailed 66-items semi-structured retrospective questionnaire Supplementary Information 4) and therapy. MIDAS and HIT6 scales were administered to each patient to measure migraine-related disability. Diagnoses of primary headache were codified according to International Criteria of Headache Disorders 3rd edition (ICHD3) criteria^16^. Possible correlations between migraine and neuromuscular phenotype or genotype were investigated. All patients had given written informed consent to the scientific use of their clinical data, which have been treated in a completely anonymous way. We excluded patients aged < 16 years or with severe cognitive decline and no reliable caregiver that made impossible the administration of the semi-structured interview, patients with intracranial space-occupying lesion or with a history of recent (less < 1 year) stroke.

### Statistical analyses

Results were displayed as mean and standard deviation (SD) or median and range when appropriate. Age at headache onset was reported using the Kaplan–Meier method, and compared between genotype/phenotype groups by Cox regression. Distributions of quantitative and ordinal variables among two or more groups were compared with the Wilcoxon-Mann–Whitney or Kruskal–Wallis tests, respectively. The effect of several risk factors on the onset of migraine was evaluated by multivariate logistic regression model. Statistical significance was set at *p* < 0.05. Analyses were performed with R v.3.5.3.

## Supplementary Information

Supplementary Information.
