# Supplementary material for: The relevance of migraine in the clinical spectrum of mitochondrial disorders
Source: Sci Rep. 2022 Mar 10;12:4222. doi: 10.1038/s41598-022-08206-z (PMC8913605; doi:10.1038/s41598-022-08206-z)
Supplement: Supplementary file 1 — Supplementary Information. [file 41598_2022_8206_MOESM1_ESM.docx]

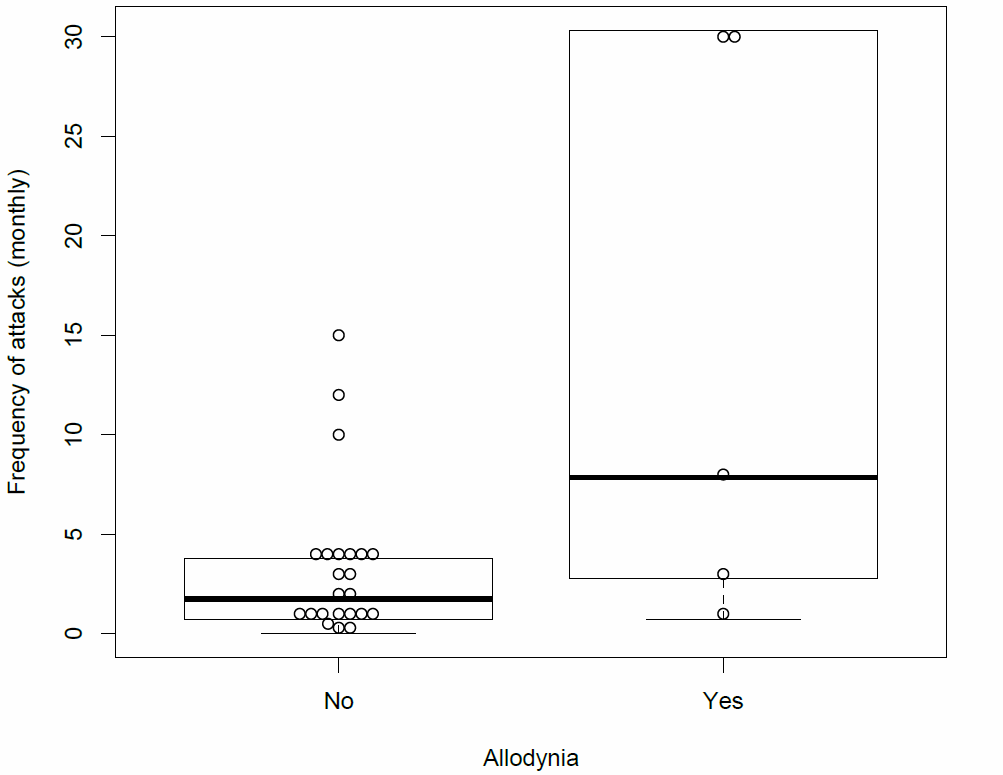


Supplementary Information 1: Box plot showing the association between allodynia and the number of migraine days per month


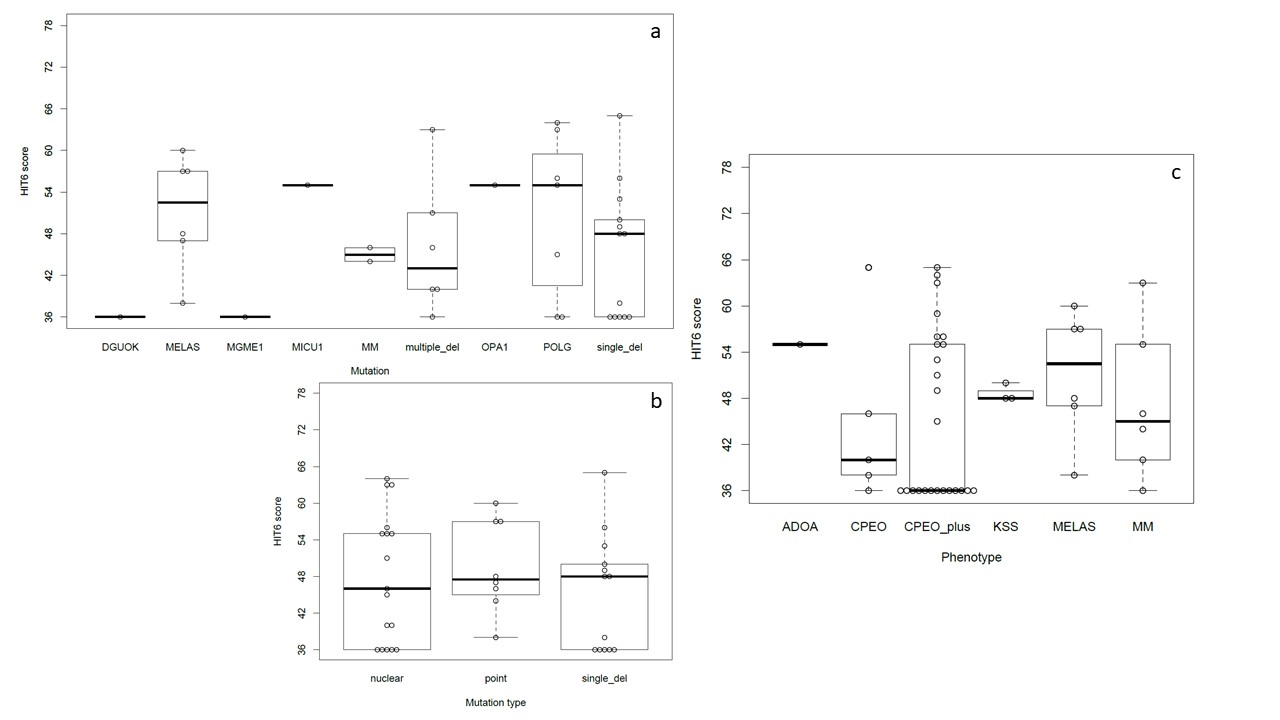


Supplementary Information 2: Box plot of HIT6 scores across the different mitochondrial genotypes (a), across the different type of mutation (b) and across the different neuromuscular phenotypes. Horizontal bars represent the mean values. Boxes include mean +/-25% of data. Abbreviations: DGUOK= deoxyguanosine kinase; MELAS= mitochondrial encephalopathy, lactic acidosis and stroke-like episodes; MGME1= mitochondrial genome maintenance exonuclease 1; MICU1= mitochondrial calcium uptake 1; MM= other point mtDNA mutations; multiple_del: multiple mtDNA deletions; OPA1= OPA1 mitochondrial dynamin like GTPase; POLG= DNA polymerase gamma; single_del= single mtDNA deletion. ADOA= autosomal dominant optic atrophy; CPEO= chronic progressive external ophthalmoplegia; KSS= Kearns-Sayre syndrome; MELAS= mitochondrial encephalopathy, lactic acidosis and stroke-like episodes; MM=mitochondrial myopathy.


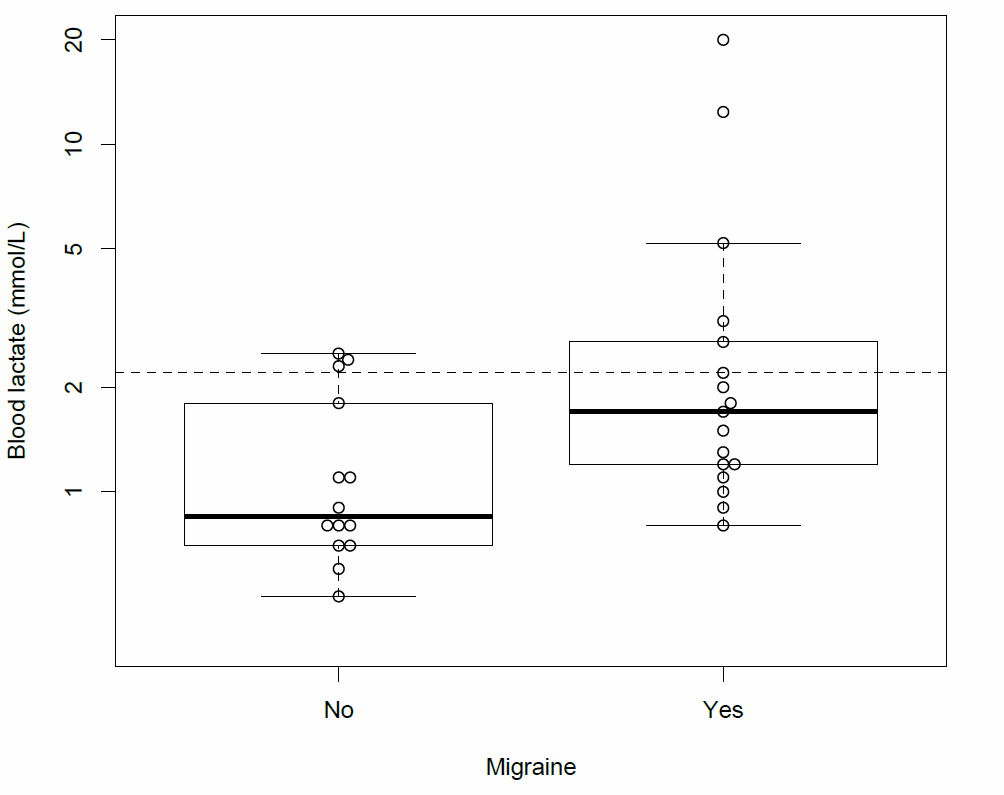


Supplementary Information 3: Box plot of blood lactate levels among patients without migraine (on the left) or with migraine (on the right). Dashed line indicate the upper limit of normality of our laboratory. Note the association between higher lactate levels and the diagnosis of migraine


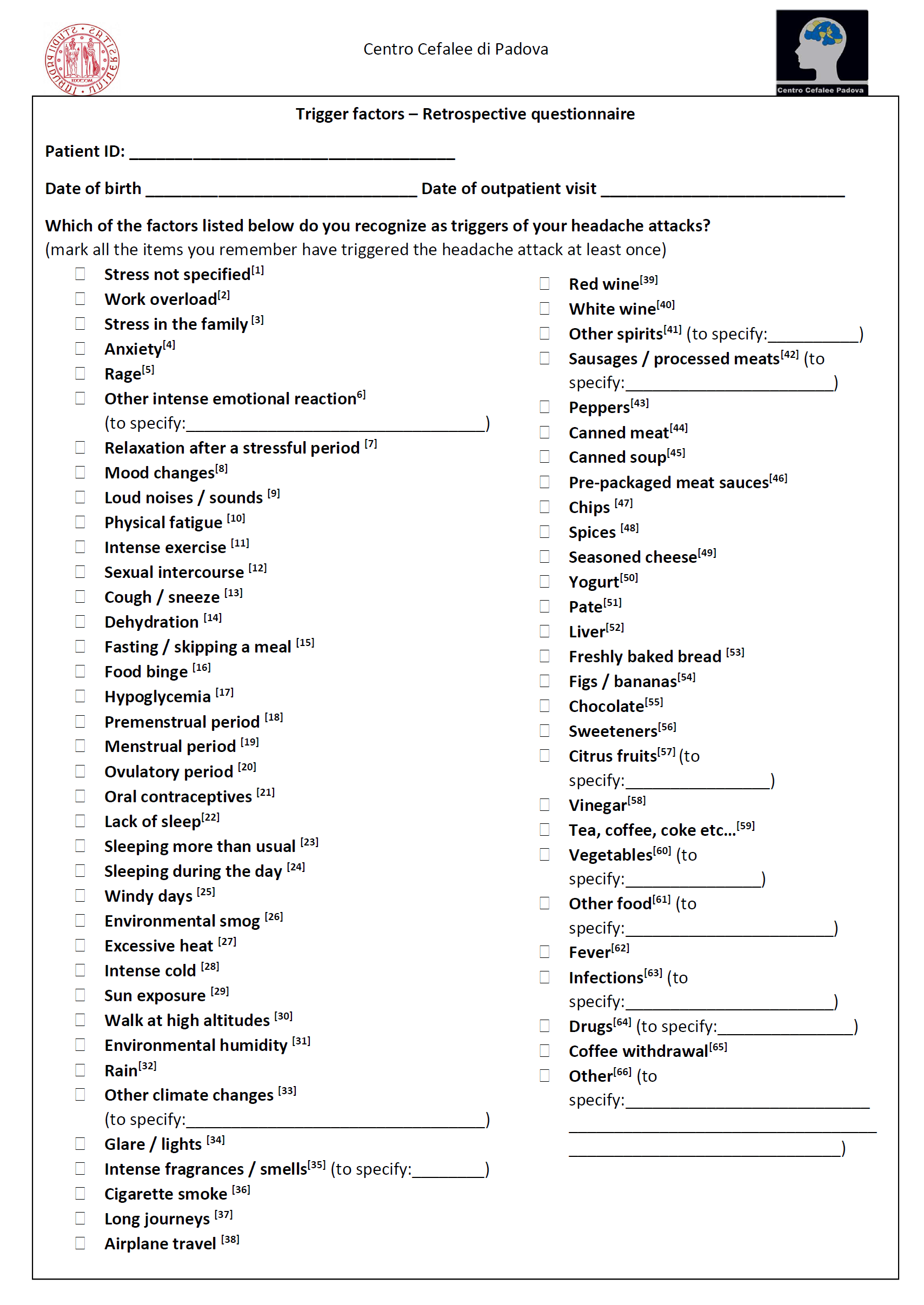


Supplementary Information 4: Retrospective semi-structured trigger questionnaire. The patient was asked to fill in the form, composed of 66 items, checking (X) those boxes corresponding to trigger factors he/she recognizes relevant for his/her migraine attacks.
